# Distributed Optical Measurement System for Plate Fin Heat Exchanger

**DOI:** 10.3390/s23063047

**Published:** 2023-03-11

**Authors:** Huajun Li, Xiao Yang, Baoliang Wang, Haifeng Ji

**Affiliations:** College of Control Science and Engineering, Zhejiang University, Hangzhou 310027, China; hjli@hdu.edu.cn (H.L.); xiao711@zju.edu.cn (X.Y.); hfji@zju.edu.cn (H.J.)

**Keywords:** distributed optical measurement, twin-core optical fiber, plate fin heat exchanger, flow boiling, mini-channel

## Abstract

The acquirement of the flow information in plate-fin heat exchanger (PFHE) is limited by its metal structure and complex flow condition. This work develops a new distributed optical measurement system to obtain flow information and boiling intensity. The system utilizes numerous optical fibers installed at the surface of the PFHE to detect optical signals. The attenuation and fluctuation of the signals reflect the variation of the gas-liquid interfaces and can be further used to estimate the boiling intensity. Practical experiments of flow boiling in PFHE with different heating fluxes have been carried out. The results verify that the measurement system can obtain the flow condition. Meanwhile, according to the results, the boiling development in PFHE can be divided into four stages with the increase in the heating flux, including the unboiling stage, initiation stage, boiling developing stage, and fully developed stage.

## 1. Introduction

Plate fin heat exchanger (PFHE) is a kind of heat exchange equipment with a compact structure, high heat transfer efficiency, and flexibility [1,2,3]. It is suitable for applications with a large difference in heat transfer coefficients on both sides, which has been widely used in petrochemical, aerospace, deep low temperature, and other fields [2]. PFHE has significant advantages in using heat energy, recovering waste heat, saving raw materials, reducing cost, and some special applications [4].

The research on heat transfer and flow characteristics of PFHE has attracted much attention from academic researchers, which is important for the investigation of the working mechanism and exchanger optimization. The numerical simulation technology based on computational fluid dynamics (CFD) is a widely used approach to obtain the flow information inside the heat exchanger [5,6,7]. Many scholars have carried out solid simulation research on the heat exchanger based on CFD to reveal the effects of the header, fin, and different working conditions on the heat transfer efficiency, flow distribution, and flow boiling performance of PFHE [8,9,10]. Abed [11] uses numerical studies to predict flow boiling kinetics, heat and mass transfer, and pressure drop in corrugated plate heat exchangers. Their model shows good agreement with the experimental data and the values obtained from the available correlations of the two-phase heat transfer coefficient and pressure drop. Zhu and Haglind [12] performed numerical fluid dynamics simulations of adiabatic water-air flow to develop a flow regime map, and the transitions have been discussed. They also predicted the pressure drop and void fraction, which has good agreement with the empirical formula. However, numerical simulation simplifies the flow based on some governing equations and solves the problem by finite-volume based method, finite element method, or a finite different method, etc. The disagreement between numerical simulation and practical conditions is inevitable [13].

Experimental methods can be used to obtain practical information on the flow inside the PFHE, which mainly includes intrusive and non-intrusive methods. Thermocouple is one of the typical intrusive sensors to measure the local temperature. Wang et al. [9] utilized eight T-type thermocouples to measure the local temperature, which is positioned at three axial stations along the inner pipe. The outside surface temperature of the pipe is then derived from the measured ones. Based on this, they investigated the influence of flow oscillation on heat transfer. An infrared (IR) camera is another feasible method to measure the temperature. Compared with the thermocouple, an IR camera can obtain the temperature field of the whole surface. Farahani et al. [14] utilized an IR camera to measure the local wall temperature and further calculated the flow boiling heat transfer coefficient. The influence of heat flux, mass flux, saturation temperature, steam quality, and inlet conditions on the flow boiling heat transfer coefficient were also investigated. To obtain the two-phase flow distribution in the header of PFHE, Tu and Wu [15] designed a PFHE with a visualization window, and the fluids were tagged with Rhodamine B and olive oil. They used three high-resolution CCD cameras to measure the velocity fields of the gas and liquid phase simultaneously by PIV and LIF.

The existing experimental methods have been widely used to provide flow information. However, some of the methods are intrusive and can only provide local information at some specific locations. In addition, the rest methods require visualization access, which will perturb the flow condition and the heat transfer performance.

In order to obtain a holistic view of boiling conditions and not perturb the flow in PFHE, this research develops a distributed optical measurement system. This system utilizes several twin-core optical fibers to detect the optical signals through some thread holes on the surface of PFHE, which reflect the flow inside the heat exchanger. By analyzing the fluctuation of the light intensity, the distribution of the two-phase flow and the intensity of the flow boiling in the heat exchanger are estimated. Meanwhile, the development of the flow boiling with different heat fluxes is also investigated.

## 2. Distributed Optical Measurement System

**Figure 1:**
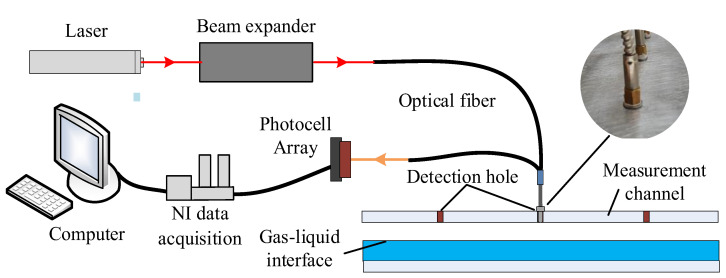
The setup of the distributed optical measurement system.

The distributed optical system developed in this work, as shown in Figure 1, includes a laser source, a beam expander, a computer, a data acquisition unit, a photocell array, and optical fibers.

A 300 mW red laser source with a wavelength of 632 nm is used as the laser source. The beam expander is used to adjust the parallel laser to the appropriate diameter. The optical fibers used in this work have two cores. The laser enters the PFHE through one core of the fiber and is absorbed, reflected, and refracted by the fluids and the interfaces. Then the laser exits through the other core of the fiber, and the outgoing laser is detected by the photocell array (Vishay BPW34). The intensity and fluctuation of the outgoing light signals reflect the fluids, gas-liquid interface, and their variation. The light signals are then collected by the NI acquisition unit and stored in the computer. Detection holes with a diameter of less than 3 mm are drilled at the surface of the measurement channel, which is used to install the optical fibers. Due to proper design and installation, the optical fiber is non-intrusive to the flow inside the channel, and hence the perturbation to the flow can be neglected.

**Figure 2:**
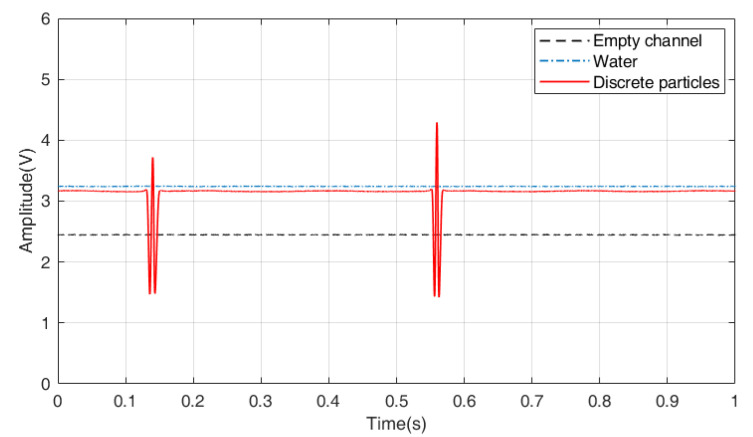
Optical signals of different flow conditions.

To better reveal the mechanism of light attenuation, preliminary experiments with the two-phase flow are carried out, where the gas phase is replaced by plastic particles, and the liquid phase is water. As shown in Figure 2, when the channel is empty, the laser is only affected by the reflection and absorption of the surface of the channel, which is invariable. Hence the signal is stable with no fluctuation, and the amplitude is 2.4 V. When the channel is full of water, the optical signal is also stable while the amplitude increases to 3.2 V. The increment of the amplitude can be explained by the divergence of the laser. From optical fiber to air, the laser is refracted and has a large divergence angle. While from fiber to water, the laser is less diverged, and hence the reflected laser has larger intensity.

As shown in Figure 2, the optical signal has obvious fluctuation when a plastic particle approaches the measurement field. The movement and number of the individual plastic particles can be easily determined according to the fluctuation.

**Figure 3:**
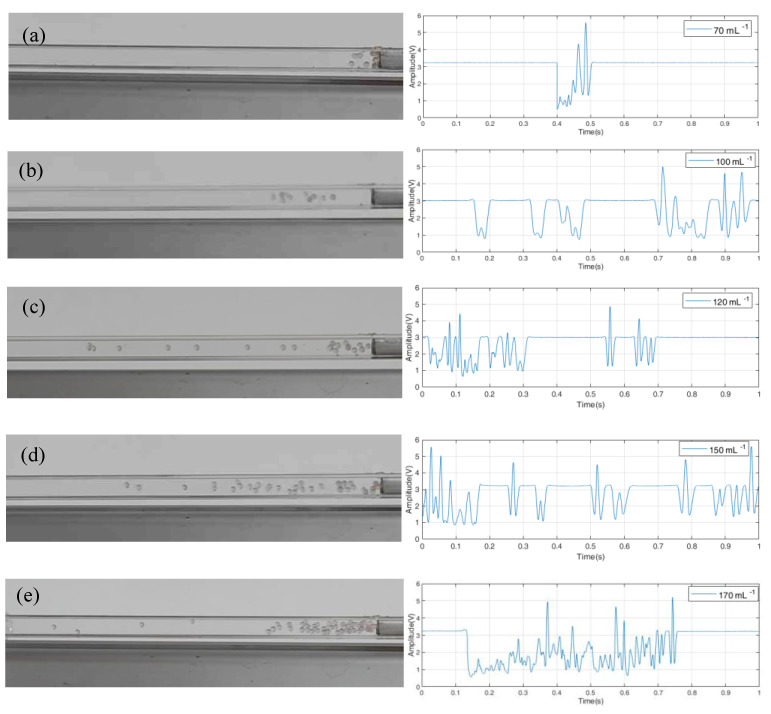
Optical signals of two−phase flow with different concentrations: (**a**) 70/mL; (**b**) 100/mL; (**c**) 120/mL; (**d**) 150/mL; (**e**) 170/mL.

The experiment with different concentrations of plastic particles is further carried out. The flow rate of the liquid is 100 mL/min for all the cases, while the concentration of the plastic particles ranges from 70/mL to 170/mL. As shown in Figure 3a, the particles start to cluster, and corresponding signals display irregular variation. The movement and number of the particles can no longer be determined specifically, as in Figure 2. The clustering and irregular variation become much worse with the increase in the concentration, as shown in Figure 3b–e.

The optical signals are further analyzed in the frequency domain. The transformation can be calculated by:

$$X\left(f\right)=\int_{-\infty }^{\infty }e^{-i2\pi ft}x\left(t\right)dt$$

where $x\left(t\right)$ represents a temporal signal and $X\left(f\right)$ is its frequency-domain signal. While its Power Spectral Density (PSD) in decibel(dB) can be defined as:

$$S\left(f\right)=10log_{10}\left(\underset{T\to \infty }{\lim}\frac{1}{T}{\left|X\left(f\right)\right|}^{2}\right)$$

**Figure 4:**
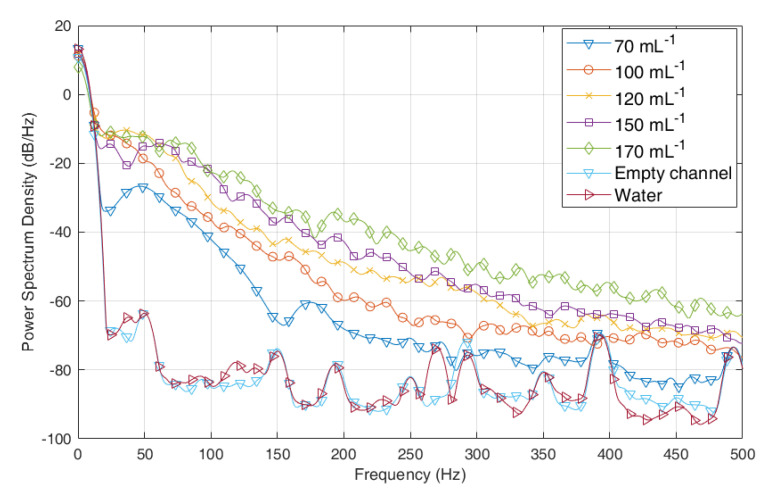
Power Spectral Density of the optical signals in decibels.

Figure 4 displays the PSD curves of the seven signals. For the signals of water and empty channel, the PSD curves have a large amplitude in the low-frequency zone while having a low amplitude in the high-frequency zone. Compared with these two curves, the PSD curve with a plastic particle concentration of 70/mL has a larger amplitude in the high-frequency zone, especially from 25 to 260 Hz. With the increase in the particle concentration, the PSD curve rises to a higher level. The signal with the largest concentration locates at the topmost.

**Figure 5:**
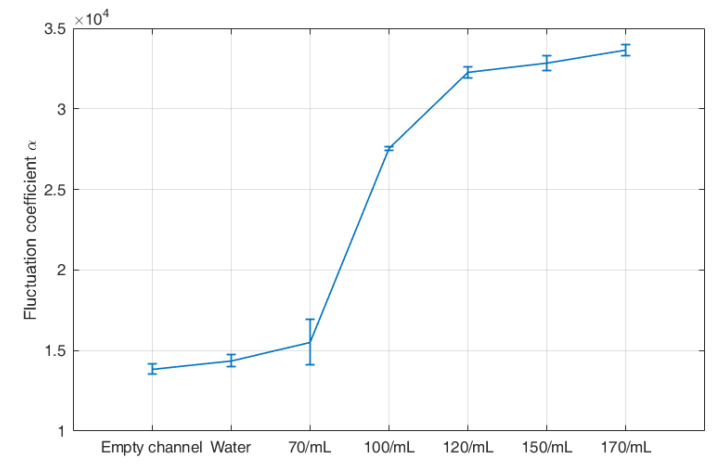
Fluctuation coefficient of the two-phase flow with different concentrations of plastic particles.

In order to quantify the fluctuation degree and reflect the flow condition, a coefficient is defined as:

$$\alpha =\int_{f_{min}}^{f_{max}}\left\{S\left(f\right)-S_{0}\right\}df$$

where, $f_{min}$ is 0 Hz and $f_{max}$ is 500 Hz. $S_{0}$ is set as −100 dB. Figure 5 displays the fluctuation coefficients of all seven signals. The results illustrate that the signals of water and empty channel have the lowest value, which is only 1.39 × 10^4^ and 1.44 × 10^4^. The signals of the flow with plastic particles have much larger values, ranging from 1.55 × 10^4^ to 3.36 × 10^4^. Meanwhile, the signals of the flow with larger concentration have larger values, which verifies the feasibility of the parameter α reflecting the fluctuation degree of the optical signals.

## 3. Experimental Setup of Flow Boiling in PFHE

**Figure 6:**
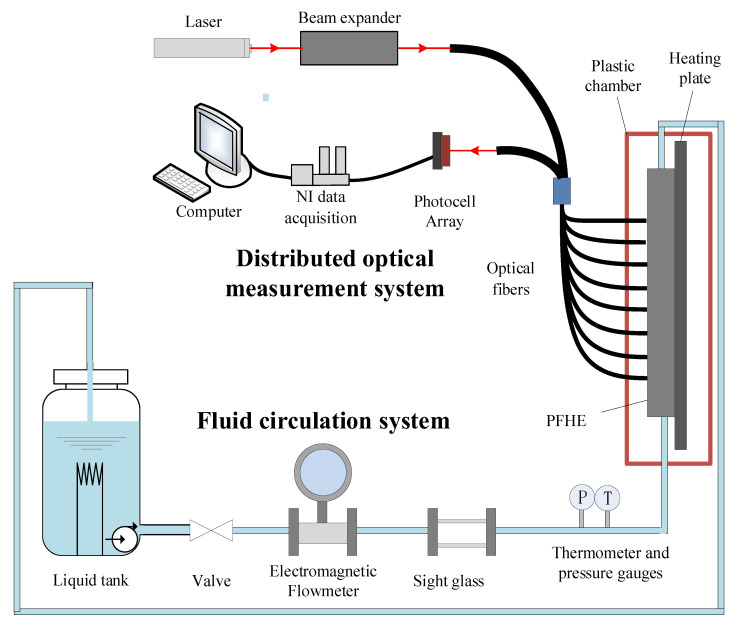
Experimental setup of the blow boiling in PFHE.

In order to verify the effectiveness of the proposed measurement system; an experimental setup is developed to induce flow boiling in PFHE and obtain the flow information. As shown in Figure 6, the fluid circulation system includes a liquid tank, an electromagnetic flowmeter, a visualization window, a heating plate, thermometer and pressure gauges, and a PFHE test piece.

The liquid tank used in this work has a volume of 25 L and can pump liquid out with a maximum flow rate of 250 L/h. The liquid tank can also pre-heat the liquid. The electromagnetic flowmeter is used to measure the flow rate, with DN 15 and an accuracy of 0.3%. The visualization window is a transparent and plastic channel, which allows the minoring of the flow condition. The copper heating plate is used to induce flow boiling, which is installed close to the PFHE. The maximum heating power of the heating plate is above 8000 W. Thermal grease is applied between the heating plate and PFHE for better heat conduction. The heating plate and the PFHE are placed in a plastic chamber for thermal insulation.

**Figure 7:**
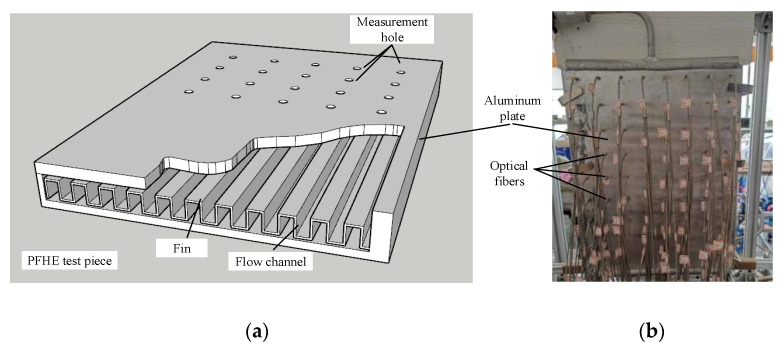
The diagram of the PFHE: (**a**) Structural sketch; (**b**) Practical installation.

**Figure 8:**
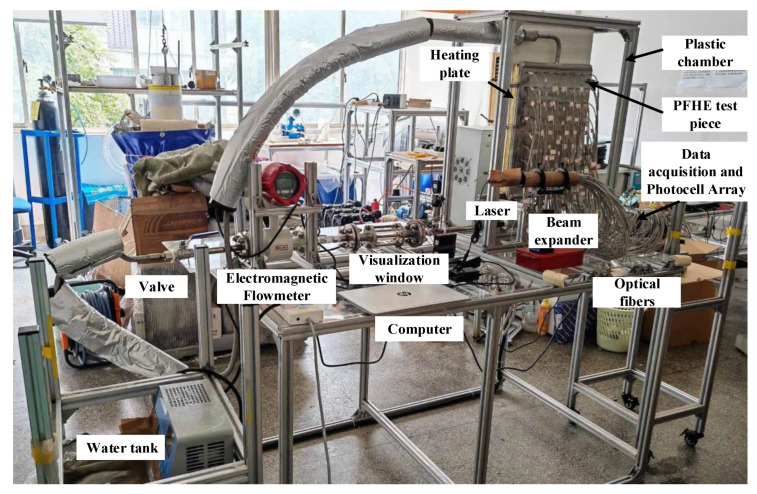
Laboratory implementation of the experimental setup of flow boiling in PFHE.

The PFHE test piece used in the experiment is aluminum with no deflector. The size of the test piece is 350 mm × 450 mm × 30 mm. As shown in Figure 7a, the inner flow channels of the heat exchanger are made of uniformly perforated fins and bent. The flow channel is 3.5 mm wide and 5.7 mm deep. The heat exchanger is placed vertically, and liquid flows upwards. In order to obtain the flow information of the whole heat exchanger, several measurement holes are drilled through the outer surface of the test piece (the other surface is attached to the heating plate), and 56 optical fibers are installed uniformly on the surface of the heat exchanger. The diameter of the measurement hole is less than 3.0 mm, and the disturbance caused by the measurement holes and optical fibers is neglected. Figure 7b displays the practical installation of the PFHE with the optical fibers. Figure 8 displays the laboratory implementation of the developed experimental setup.

## 4. Results and Discussion

The flow boiling experiments with different heating conditions have been carried out, and the characteristic of the flow has been detected and analyzed. Table 1 lists all the conditions carried out in this work. The heating temperature of the heating plate ranges from 95 to 130 °C, and the heat flux ranges from 2.728 to 23.551 kW/m^2^. The flow rate is 230 L/h when the heating temperature is 95 °C. At the same time, the flow rate is affected by the flow boiling. When the heating temperature is 130 °C, the flow rate is decreased to 42 L/h.

**Figure 9:**
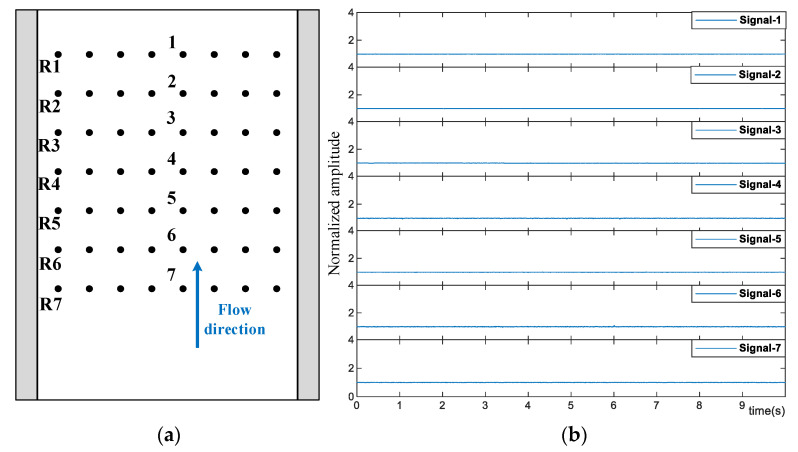
The selected optical fibers (**a**) and their optical signals (**b**) with a heating temperature of 90 °C.

**Figure 10:**
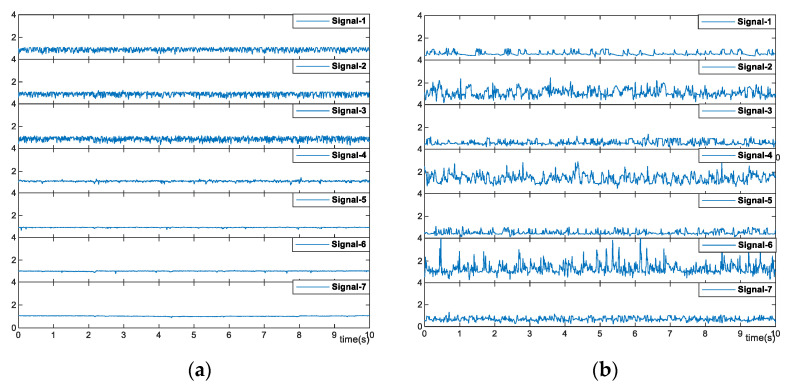
Optical signals of the selected optical fibers with a heating temperature of (**a**) 115 °C and (**b**) 130 °C.

Figure 9a illustrates the arrangement of the 56 optical fibers with seven rows and eight columns. Figure 9b and Figure 10 display a group of signals obtained by the central optical fibers, with a heating temperature of 90, 115, and 130 °C. Since the amplitude of the signals is affected by the installation and the intensity of the incident laser, all the signals are normalized by their amplitude of un-boiled condition, respectively. It can be seen from Figure 9b that when the heating temperature is 90 °C, all seven signals remain stable with no obvious fluctuation, and the flow inside the PFHE stays single-phase.

When the temperature increases to 115 °C, the seven signals display much difference. As shown in Figure 10a, the bottom three signals are stable with minor fluctuation. While signal-4 has obvious fluctuation, indicating the variation of the interface and the initiation of the flow boiling. The fluctuations become more obvious for the top three signals. The flow condition can be easily inferred that from signal-7 to signal-5, the flow is heated and stays single-phase. Then, the flow boiling starts around signal-4, and the flow becomes two-phase. From signal-3 to signal-1, the flow boiling becomes more intense, which leads to the obvious fluctuation of the signals.

Figure 10b illustrates the signals of the flow with a heating temperature of 130 °C. All seven signals have obvious fluctuation, indicating the flow boiling all over the detected district. In this case, the signals have much more severe fluctuation, where the amplitude of the fluctuation can be up to 300%.

**Figure 11:**
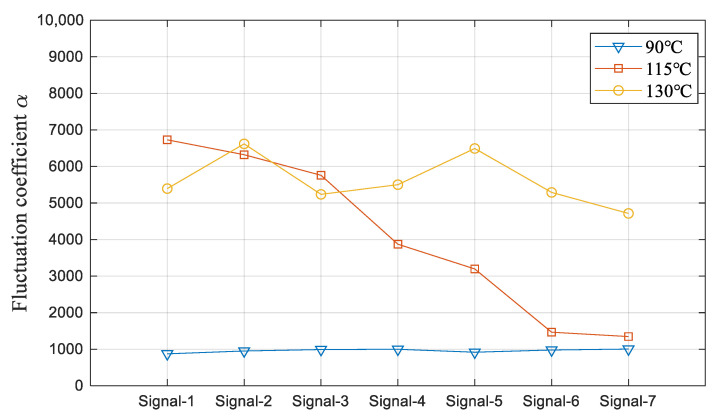
Fluctuation coefficients of the signals with a heating temperature of 90, 115, and 130 °C.

Figure 11 displays the fluctuation coefficient of all seven signals with the heating temperature of 90, 115, and 130 °C, which clearly illustrates the development of the flow boiling in PFHE.

**Figure 12:**
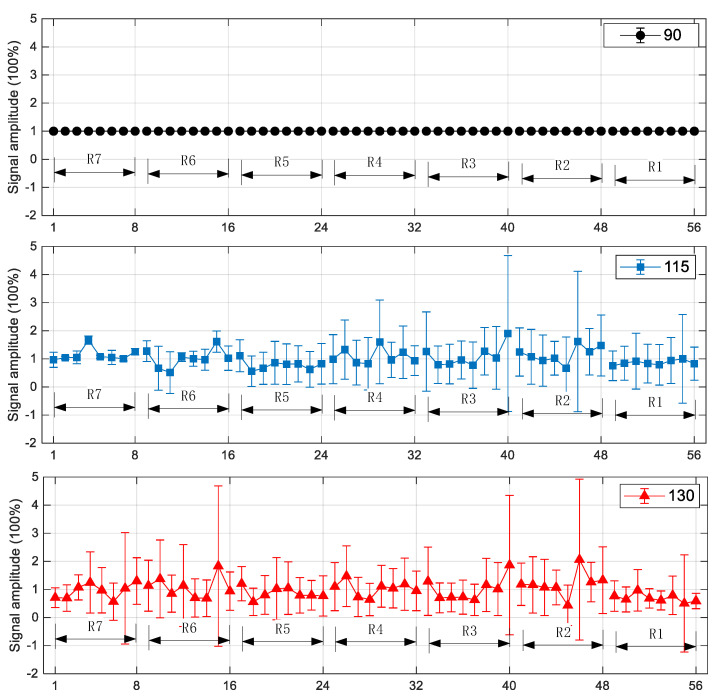
Means values and deviations of the signals with heating temperatures of 90, 115, and 130 °C.

Figure 12 displays the mean values and their deviation of all the signals with the three heating temperatures. It can be seen from the figure that the signals with a heating temperature of 90 °C have identical amplitude and negligible fluctuation; When the heating temperature is 115 °C, the deviation of the signals has much different: for the signals in R7 and R6, the deviations are relatively small; from R5 to R1, the deviations become significant, with the maximum up to 270%. When the heating temperature reaches 130 °C, all 56 signals have comparable and obvious deviations.

**Figure 13:**
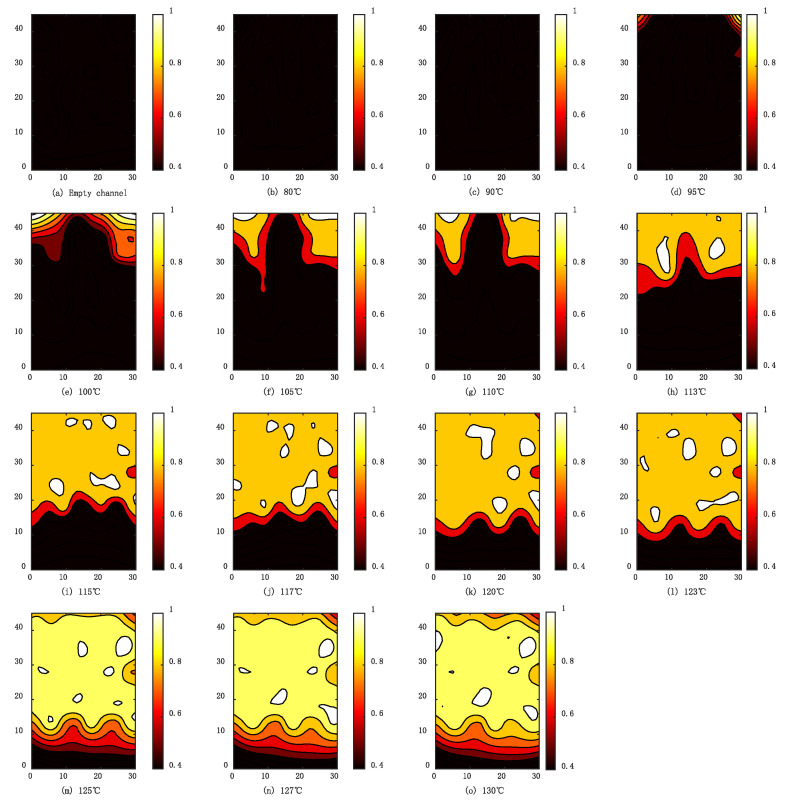
Visualization of the flow boiling in PFHE with different heating temperatures: (**a**) Empty channel; (**b**) 80; (**c**) 90; (**d**) 95; (**e**) 100; (**f**) 105; (**g**) 110; (**h**) 113; (**i**) 115; (**j**) 117; (**k**) 120; (**l**) 123; (**m**) 125; (**n**) 127; (**o**) 130.

In order to obtain the overall estimation of the flow condition inside the PFHE test piece, the fluctuation coefficients of all 56 signals under different heating temperatures are calculated and normalized by the maximum coefficient of all the conditions and signals. Meanwhile, biharmonic spline interpolation is used to visualize the results, as shown in Figure 13.

When the PFHE is empty, or the heating plate is below 95 °C, the fluctuation coefficient of the whole surface is below 0.4, as shown in Figure 13a–c. In these conditions, the fluid maintains a single phase, and the light intensity signals are stable with neglectable fluctuation.

When the temperature of the heating plate reaches 95 °C, as shown in Figure 13d, the α of some areas is above 0.4, which indicates the initiation of flow boiling. The boiling can be first observed at the upper two corners of the exchanger. Due to the structure of the PFHE, the flow velocity field is non-uniform, and the region at the corners has a much lower velocity, which can be the reason for the first initiation of boiling at these regions.

As the temperature continues to rise, the coefficient becomes larger, and the boiling inside the heat exchanger is more intense. The boiling area begins to expand downwards. In these cases, the left and right region of the heat exchanger boils much earlier than the central region, which can be seen in Figure 13e–h. This phenomenon can also be explained by the non-uniformity of the flow velocity. Since the fluid velocity of the left and right regions of the heat exchanger is slower than the velocity in the central region, the fluid in these regions is heated longer and hence boils earlier.

When the temperature increases from 115 to 123 °C, the boiling area expands towards the inlet of the heat exchanger. In this stage, the boundary between single-phase flow and flow boiling area is horizontal, and the influence of velocity non-uniformity on flow boiling is less obvious. The fluctuation coefficient α is up to 0.8.

As shown in Figure 13m–o, when the temperature exceeds 125 °C, the α reaches its maximum, and the boiling region covers the majority of the PFHE and stops expanding. In this stage, the boiling at the top of the heat exchanger is less intensive, with α of only 0.8, compared with the other boiling region. Due to the continuously intensive boiling of the fluid through the exchanger, the vapor fraction is increased, and hence the boiling at the region close to the outlet is restrained.

**Figure 14:**
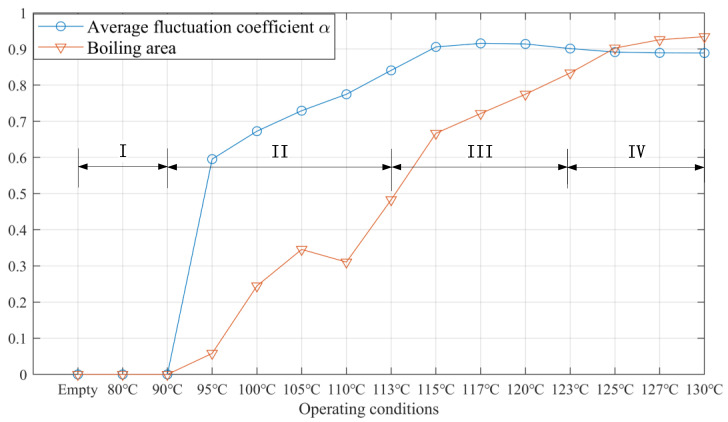
Boiling area and average fluctuation coefficient (normalized) of the considered heating conditions, divided into four stages.

Figure 14 displays the curves of the boiling area and the average fluctuation coefficient. The boiling area is defined as the percentage of the area with a fluctuation coefficient larger than 0.45, and the average fluctuation coefficient is the average value in the boiling area. According to the results, the boiling conditions with different heating temperatures can be divided into four stages:

(1) when the temperature is below 95 °C, the flow is single-phase with α equals 0. In this stage, the signals have no obvious fluctuation;
(2) the flow starts to boil at the two corners of the exchanger first when the temperature is above 95 °C. In addition, the boiling area expands downwards quickly. When the heating temperature is 95 °C, the boiling area is only 6%, while when the temperature is 113 °C, the boiling area reaches 48%. The average fluctuation coefficient also increases quickly, from 0.60 at 95 °C to 0.84 at 113 °C.
(3) in stage III, the boiling area has a smaller increment. From 115 to 123 °C, the area only increases from 67% to 83%. At the same time, the average coefficient stays in the range of 0.90 to 0.91.
(4) when the temperature is above 125 °C, the boiling covers more than 90% of the whole exchanger and nearly stops expanding. Meanwhile, the average coefficient has minor variation.

**Figure 15:**
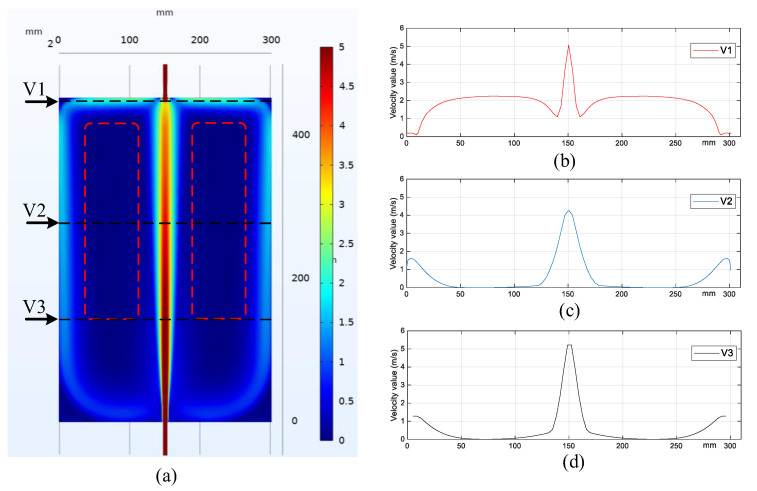
Simulation results of the PFHE: velocity field (**a**) and velocity values of the horizontal lines with a distance of 150 mm (**b**), 300 mm (**c**), and 400 mm (**d**) from the inlet.

Numerical simulation with COMSOL has been conducted to reveal the flow velocity field inside the PFHE. The geometry size of the simulated PFHE model is 350 mm × 450 mm × 30 mm, and heat transfer is not involved. The flow rate is 230 L/h. Figure 15 displays the simulation result of the velocity field and the values at three horizontal lines. As shown in Figure 15a, the central axis has the largest velocity due to the absence of a deflector, which can be up to 5.2 m/s at the inlet and outlet. The flow close to the left and right boundary has a relatively larger velocity compared with the surrounding area, as shown in Figure 15c,d. Two areas with nearly zero velocity have been marked in Figure 15a. Meanwhile, the upper two corners of the PFHE also have low velocities, as shown in Figure 15b. The simulation results have verified that the maldistribution of the velocity has good agreement with the development of the flow boiling. Since the upper two corners have low velocity, the boiling initiates firstly, as shown in Figure 13d; Since the marked region has nearly zero velocity, the boiling expands into this region earlier than the rest part, as shown in Figure 13f,g.

## 5. Conclusions

In this paper, the distributed optical measurement system is developed to visualize the intensity of flow boiling in a PFHE. The system utilizes 56 two-core fibers to monitor the boiling intensity, which is mounted uniformly on one surface of the PFHE. The distribution and development of the flow boiling in the heat exchanger are experimentally studied based on the measurement system. Meanwhile, the fluctuation coefficient is introduced to reflect the intensity of flow boiling. Experiment results show that the measurement system can effectively obtain information on the flow boiling.

According to the experiment results, the boiling in the heat exchanger can be mainly divided into four stages, including the unboiling stage, initiation stage, boiling developing stage, and fully developed stage: (1) when the temperature is below 95 °C, the flow is single-phase and un-boiled; (2) the flow starts to boil at the two corners of the exchanger firstly when the temperature is above 95 °C. In addition, the boiling area expands downwards non-uniformly when the temperature increases; (3) when the temperature is in the range from 115 to 123 °C, the boiling area is further expanded, while the overall boiling intensity stays the same; (4) the boiling stops expanding when the temperature is above 123 °C, and the boiling of the region close to the outlet is restrained. Numerical simulation has verified that the maldistribution of the velocity field is the main reason for the first initiation of the boiling at the upper two corners and the non-uniform development.

The experimental results verify the effectiveness of the distributed optical measurement system, while further research concerning the mechanism and the non-uniformity of boiling need to be investigated. Meanwhile, the influence of flow boiling on the heat transfer coefficient will be further investigated. In addition, the application of the developed measurement system in industrial and practical fields will also be implemented.

## Figures and Tables

**Table 1:** The heating condition and flow rate of the experiments.

Heating Temperature (°C)	Heat Flux (kW/m^2^)	Flow Rate (L/h)
95	2.728	230
100	2.866	225
105	4.355	210
110	6.736	130
113	8.000	110
115	9.140	100
117	10.713	85
120	12.847	80
123	15.833	75
125	17.7412	60
127	19.947	50
130	23.551	42

## Data Availability

Data is unavailable due to privacy policy.
